# Supervised Deep Learning for Detecting and Locating Passive Seismic Events Recorded with DAS: A Case Study

**DOI:** 10.3390/s24216978

**Published:** 2024-10-30

**Authors:** Emad Al-Hemyari, Olivia Collet, Konstantin Tertyshnikov, Roman Pevzner

**Affiliations:** Centre for Exploration Geophysics, Curtin University, GPO Box U1987, Perth, WA 6845, Australia; emad.al-hemyari@postgrad.curtin.edu.au (E.A.-H.); olivia.collet@curtin.edu.au (O.C.); konstantin.tertyshnikov@curtin.edu.au (K.T.)

**Keywords:** DAS, passive seismic, induced seismicity, deep learning

## Abstract

Exploring shallow mineral resources requires acquiring denser seismic data, for which Distributed Acoustic Sensing is an effective enabler and relevant to mining operations monitoring. Passive seismic can be of interest in characterizing the subsurface; however, dealing with large amounts of data pushes against the limits of existing computational systems and algorithms, especially for continuous monitoring. Hence, more than ever, novel data analysis methods are needed. In this article, we investigate using synthetic seismic data, paired with real noise recordings, as part of a supervised deep-learning neural network methodology to detect and locate induced seismic sources and explore their potential use to reconstruct subsurface properties. Challenges of this methodology were identified and addressed in the context of induced seismicity applications. Data acquisition and modelling were discussed, preparation workflows were implemented, and the method was demonstrated on synthetic data and tested on relevant seismic monitoring field dataset from the Otway CO_2_ injection site. Conducted tests confirmed the effects of time shifts, signal-to-noise ratios, and geometry mismatches on the performance of trained models. Those promising results showed the method’s applicability and paved the way for potential application to more field data, such as seismic while drilling.

## 1. Introduction

Distributed Acoustic Sensing (DAS) enables efficient seismic data acquisition for a range of geophysical applications, including vertical seismic profiling (VSP), permanent reservoir monitoring, earthquake and microseismicity detection, and continuous passive seismic recording [1,2,3,4,5]. Using low-cost fiber optic cables, typically used for telecommunication, as distributed sensor arrays enables permanent installations of large sensor arrays in challenging surface or borehole settings [6,7,8]. They do not contain electronic or mechanical components; thus, they are immune to electrical and electromagnetic interferences and do not require recalibration compared to conventional seismic sensors. However, their primary advantage lies in their flexibility to provide dense spatial sampling [9]. Coupled with continuous recording, they enable recording wideband seismic signals, but this generates large volumes of data that challenge current computational systems and algorithms [10,11]. Consequently, there is an increasing need for novel methods to analyze big data.

In the case of mineral resources exploration, surveying using active 3D seismic methods has not proliferated as much as it has in the oil and gas industry [12,13]. This is mostly due to the smaller scales and shallower depths of exploration targets. Additionally, the widespread adoption of active seismic exploration methods may still be challenging, especially in greenfield exploration, as they are perceived to have higher costs. Hence, exploration methods with lower spatial sampling and shallower penetration depths, such as drilling and non-seismic methods, have been sufficient so far [14]. However, as the demand for minerals continues to rise, we will eventually exhaust all the easier mineral targets, and exploration for deeper targets will be inevitable [15,16]. That is where active seismic may have a huge potential for deeper target exploration and imaging, especially in hard rock environments [17]. However, in the interim period, until active seismic surveys are used at larger scales, we suggest using passive seismic methods to provide cheaper alternatives. This is especially valuable as the current exploration methods rely on drilling for the most part. Drilling generates pulses of seismic energy, which can be used as passive seismic sources, replacing the need for active counterparts. The aim is to produce low-impact and high-precision greenfield exploration methods extending to brownfield exploration, mine monitoring, and safety applications [18,19].

**Figure 1:**
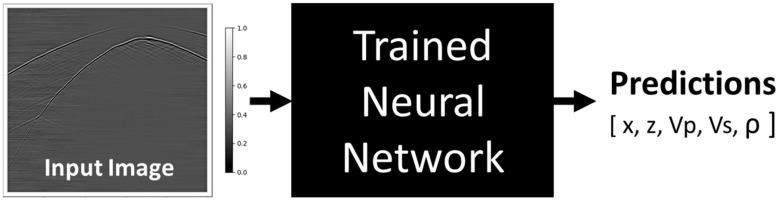
A high-level diagram demonstrating the application of supervised deep learning.

In this article, we investigate an approach based on a supervised deep-learning convolutional neural network and extend it to detect and locate passive seismic sources automatically [20,21]. After a neural network is trained, images of pre-processed field data can be fed into the neural network to output predictions of event depth and offset (z, x) and subsurface properties, including estimates of P-wave and S-wave velocities (Vp, Vs) and density (ρ) at the event location, as shown in Figure 1. These estimates can then be used to reconstruct property logs for all depths, given a sufficient number of detected events.

Implementing the supervised deep learning approach requires understanding a few inherent challenges to address them adequately. To train a neural network in a supervised manner, the first challenge is obtaining enough labelled training data. This is particularly difficult for specialized domains like seismic applications, unlike typical classification tasks (e.g., differentiating between cats and dogs), which benefit from abundant labelled data available on the web [22]. Hence, we use synthetically generated seismic data to address this challenge and prepare a sufficient training dataset. As a result, the second challenge stems from the gap between synthetically generated and field-recorded seismic data; they look completely different. Differences may be associated with, but not limited to, inaccuracies of subsurface models used for modelling, differences in acquisition geometries, differences in seismic sources, frequency bandwidths, multiple noise sources, and different signal levels in the field data.

In the following sections, this article addresses aspects related to field data acquisition and synthetic data modelling, elaborating on method implementation and data preparation workflows, followed by results, discussions, and conclusions.

## 2. Data Acquisition and Modelling

Seismic modelling is key to training neural networks using synthetically generated seismic data. The aim is to generate synthetic seismic data representative of the field data. Hence, a solid understanding of the acquired DAS data is key to achieving that goal.

### 2.1. Otway Induced Seismicity Dataset

**Figure 2:**
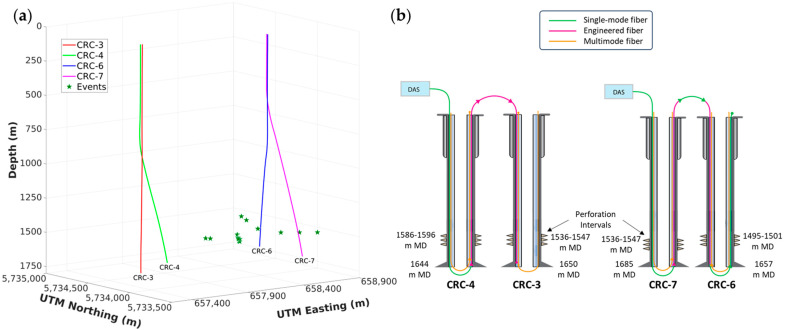
(**a**) A diagram of the four wells used to record induced events at the Otway Stage 3 CO_2_ injection monitoring site, where green stars mark event locations. (**b**) Configuration schematics of the fiber-optic cable installations.

A seismic dataset from the Otway Stage 3 CO_2_ injection monitoring project, located on the southern shores of the Australian state of Victoria, is used to demonstrate and test the methodology at a reasonable scale. Multiple wells instrumented with DAS were used to continuously record passive seismic data before and during the injection of CO_2_ into a reservoir at around 1550 m depth. Hundreds of terabytes of data were recorded from multiple wells and scanned to detect and locate induced events. The detection was done in two stages, first by using the ratio of short-time to long-time averages (STA/LTA), then with a semblance-based method using waveform cross-correlations to narrow the search. To locate the detected events, travel-time curves of P- and S-waves were analyzed first. The depths of hypocenters were estimated based on the P-wave mean apex depth, which was then used in a grid search to estimate the lateral locations based on time delays between P- and S-waves. Reasonable lateral uncertainty was demonstrated in the direction between the two well groups. Event magnitudes were also estimated [23]. Thus, the detected induced event locations are used as a reasonable ground truth to validate predictions. Specifically, we rely on DAS data from four wells (CRC-3, CRC-4, CRC-6, and CRC-7) with depths of around 1650 m, as shown in Figure 2a. The wells are slightly deviated, except for the CRC-3 well. Simplified schematics of fiber-optic installations in the four wells are shown in Figure 2b.

The CRC-4 and CRC-3 cables were spliced together, starting with CRC-4, then CRC-3, and connected to the same interrogator. Similarly, CRC-7 and CRC-6 fiber-optic cables were spliced together and connected to a second interrogator. Using the 5 m grading engineered fiber sections of the cables cemented behind the casing, passive seismic records of 30 s were acquired using DAS at a one-millisecond sampling rate and approximately 1 m spatial sampling for over two years. Silixa Carina DAS interrogators were used to produce strain-rate measurements.

### 2.2. Model Building and Modelling

**Figure 3:**
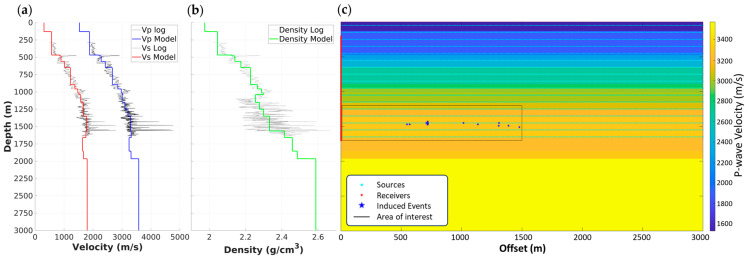
Upscaled 1D model of (**a**) P-wave and S-wave velocity and (**b**) density from vertical CRC-3 well. (**c**) An extended 2D model over 3000 m of offset with an overlay of source and receiver locations used for modelling, focusing on an area of interest around the induced event locations.

Additionally, a well log with a 1 m sampling interval, acquired in a vertical well CRC-3, was used to generate an upscaled 1D elastic properties model (P-wave and S-wave velocities and density), as shown in Figure 3a,b. The log upscaling was done by honoring major subsurface formations at the Otway site and using Backus averaging, resulting in a 1D model with 17 layers of variable thicknesses. This single 1D model represents the Otway subsurface model well and thus is sufficient to demonstrate the method, limiting the complexity of the problem. Perturbations of the 1D model are addressed briefly in the discussions as part of the problem complexity subsection. Elastic seismic modelling was subsequently performed to generate synthetic data for several passive events covering an area of interest around the reservoir level. To perform the elastic modelling, the 1D model was extended to make a 2D model spanning offsets ranging from 0 to 3000 m from the CRC-3 well, as shown in Figure 3c.

Receivers spanning a depth range of 200 to 1700 m with a 1 m spacing were used to imitate the DAS receivers in the vertical CRC-3 well. Elastic modelling was then performed for each source location using a vertical point source with an amplitude of 1 Newton, a 50 Hz central frequency, and a maximum frequency of 170 Hz, to generate a representative synthetic dataset in a reasonable amount of compute time. This source type is chosen to model primary and shear wavefields irrespective of the source mechanism. Perturbating the source mechanism is addressed briefly in the discussions as part of the problem complexity subsection. The modelling was performed using the Ocean Acoustics and Seismic Exploration Synthesis (OASES) base package, an efficient numerical modelling code for propagation in horizontally stratified media, based on the global matrix method in the frequency-wavenumber domain [24]. The total number of modelled synthetics was 5100, resulting in records with 1501 receiver channels and 5000 samples at a sampling rate of 1 millisecond. These synthetics model particle velocity, which requires converting them to strain-rate measurements to match the recorded DAS field data.

### 2.3. Field vs. Modelled Data

**Figure 4:**
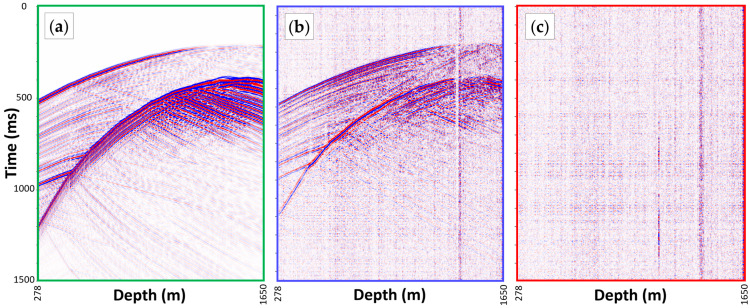
Three 1.5 s long strain-rate seismograms with matching geometries of (**a**) synthetic data for a source located at a depth of 1450 m and an offset of 720 m from CRC-3 well, (**b**) a strong induced event at an estimated depth of 1470 m and an offset of 720 m from CRC-3 well, and (**c**) a noise record from CRC-3 well.

A sample synthetic record 1.5 s long was used as a point source at a depth of 1450 m and an offset of 720 m, shown in Figure 4a. An equivalent field data seismogram of a strong induced event, recorded using DAS and covering a similar depth range, is shown in Figure 4b for comparison. The induced event has an estimated source depth of 1470 m and an offset of 720 m from the CRC-3 well. The synthetic and field data seismograms in Figure 4a,b were aligned, their geometries were matched, and a bandpass filter (6.5–13–120–170 Hz) was applied to all three records for easier comparison.

The modelling provided a general understanding of the expected waveforms, where we observed similarities demonstrating the potential use of synthetics to train neural networks and differences demonstrating the gap between synthetics and field data, as discussed earlier. Another source of differences is the variable signal-to-noise ratio (SNR) in the field data compared to clean synthetic data. A sample noise record, acquired before the injection period from the same site and with the same geometry, is shown in Figure 4c. The noise record, acquired during a quiet time of the day, shows typical DAS-related noise patterns, such as coherent horizontal striping over all channels, vertical noise stripes and spikes at a few noisy channels, and random ambient noise. Recorded noise is added to synthetics to narrow the synthetic and field data mismatch.

Furthermore, synthetic modelling carries information on travel times from a reference zero time, which is different for continuous recordings of induced events. Hence, time shifts are considered in the data preparation workflow, which is discussed in further detail in the following section.

A subset of 750 synthetics from an area of interest (AOI) surrounding the locations of the induced events was selected to control the amount of data used to demonstrate the method, as highlighted in Figure 3c. The AOI covers a depth range between 1250 and 1650 m with 100 m spacing and offsets between 10 and 1500 m with 10 m spacing.

## 3. Supervised Deep Learning

### 3.1. ResNet50 Neural Network

The proposed neural network is the ResNet50, a 50-layer Convolutional Neural Network (CNN) architecture typically used for computer vision and image recognition [25]. ResNet, which stands for residual network, addresses the problem of vanishing gradients by introducing residual blocks to improve the accuracy of the models. These residual blocks have skip connections that allow alternate routes for the gradient to pass through and ensure adequate performance of higher layers compared to lower layers of the model. In other words, the goal of training a neural network is to model a target function h(x). Adding the input x to the output of the network, i.e., adding a skip connection, forces the network to model residual function f(x) = h(x) − x rather than h(x). This is called residual learning.

**Figure 5:**
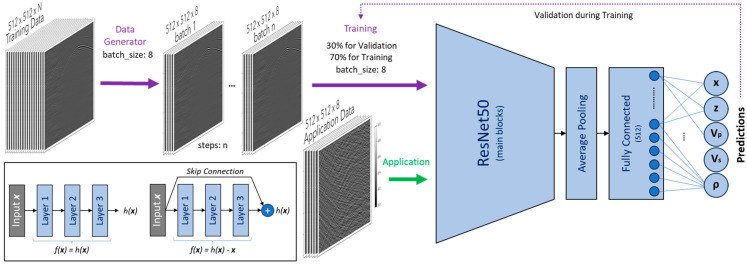
A schematic demonstrating the application of the deep-learning approach for microseismic event detection, location, and subsurface property estimation using ResNet50 architecture.

Moreover, the weights of a regular neural network are close to zero at the initialization stage, so the network only outputs values close to zero, while with a skip connection, the resulting network outputs a copy of its inputs; that is, it initially models the identity function. This is particularly useful because it speeds up the training considerably, as the target function is often close to the identity function. The neural network is set up to input 512 × 512 pixels (1 channel) images, and a few more average pooling and fully connected layers were added at the end, as shown in Figure 5. The resulting network has 24,633,093 parameters, out of which 24,579,973 are trainable.

### 3.2. Data Preparation Workflows

**Figure 6:**
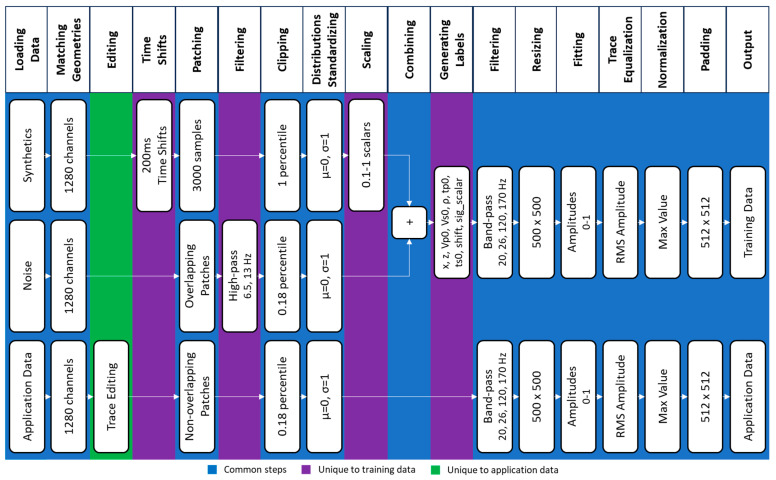
Training and application data preparation workflow highlighting common steps in blue, steps unique to training data in purple, and steps unique to application data in green.

A workflow is developed to prepare both the training and application data as required by the ResNet50 neural network. The data preparation workflow is then applied independently to the training and application data, as shown in Figure 6. The training data preparation starts with loading modelled and noise records from all wells and matching all receiver channel depths of modelled synthetics to those of the field data, which limits each record to 1280 channels per well. To simulate different onset time arrivals and extend the training dataset, each modelled synthetic is shifted in time in steps of 200 milliseconds. This results in 12,364 seismograms, 1280 channels each, which are then limited to 3000 samples per channel.

As the modelling does not consider low frequencies present in the field data, we apply a high-pass filter to the noise data to remove frequencies below 13 Hz. Hundreds of continuous records of noise data, 30 s long, were selected randomly at a period before any injection activities, in consideration of different times of the day and types of noise. These records were split into image patches 3 s long. Noise records from CRC-3, CRC-4, and CRC-6 wells were split into patches with overlapping time windows. These patches were then randomized to generate a rich noise dataset with 27,100 noise patches 3 s in length each.

The recorded noise data had a wide amplitude dynamic range; hence, we applied a 0.18-percentile clip to remove amplitude spikes at both positive and negative ends. We also apply a 1-percentile clip to the modelled data. The amplitude clipping is key to stabilizing subsequent standardization and amplitude fitting steps. Next, as both synthetics and noise patches had different ranges or scales, they were standardized to have means of zero and standard deviations of one. This standardization simplifies the combination of synthetics with noise. After that, each synthetic record was multiplied by a random scalar between 0.2 and 1 before being added to a random noise patch.

At this stage, each semi-synthetic patch was associated with a set of labels that included source offset and depth, P-wave and S-wave velocities and densities at the source location, P-wave and S-wave onset time arrivals, time shifts, and signal scalars. The remaining noise patches were also included in the training dataset but have zero labels. This was specifically intended as part of the neural network design to classify between data patches containing induced events from other patches that only contain noise, as we did not anticipate any induced events at zero depths, which in turn were not expected to yield useful subsurface information.

For the application data, the data preparation workflow is similar, as shown in Figure 6. One extra step applied to the application dataset consists of limiting the frequency bandwidth to match the bandwidth of the training data before preparing the 512 × 512 patches, which were input into the neural network. Common steps applied to the training and application datasets included data loading, geometry matching, amplitudes clipping, standardizing distributions, bandpass filtering, resizing, and fitting amplitudes between 0 and 1 before equalizing and padding. The generated training dataset consists of 12,364 semi-synthetic and 14,736 pure noise patches and their associated labels. Finally, each patch from the training and application datasets was converted to a grayscale image. This ensured that the source mechanism amplitude polarity changes were corrected for both datasets.

### 3.3. Neural Network Training

The randomized semi-synthetic dataset was used to train the neural network over 300 epochs, with 30% reserved solely for validation during the training. The training involved deriving the optimum network parameters and minimizing the mean squared error (MSE). As the dataset was relatively large for optimization in one go, the training was performed in batches of eight, and the network parameters were updated using an Adam optimizer with a linearly decaying learning rate [26]. The implementation also used data generators to feed smaller portions of the training dataset to ensure efficient GPU memory utilization. This was particularly important as we used a single NVIDIA RTX A4000 GPU on a high-end machine with 16GB GPU memory. The training took around 39.5 h to complete. Training and validation losses continued to decrease during training before stabilizing beyond 150 epochs, suggesting convergence. Similarly, the training and validation accuracies stabilized beyond 150 epochs as they continued to increase.

## 4. Results

### 4.1. Testing on Synthetic Data

**Figure 7:**
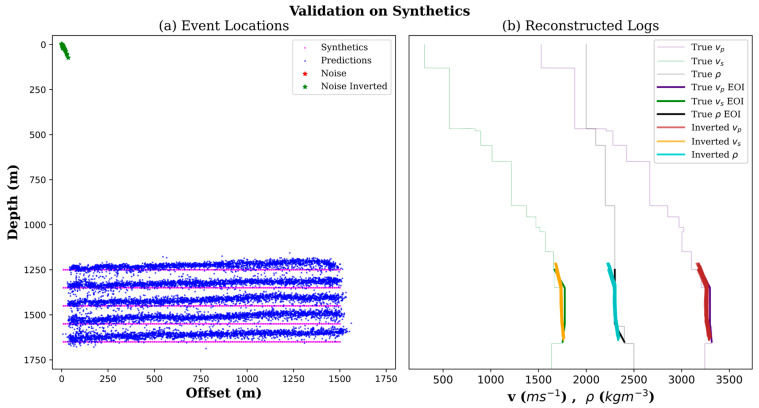
(**a**) Synthetic events location predictions compared to the ground truth locations used for training. (**b**) Reconstructed subsurface properties were overlayed on the true models.

After completing the training, the training data was fed into the neural network as an input. The aim here is to evaluate the performance of the trained network using synthetics and compare outputs against their known labels, a standard practice in machine learning. Sensitivity analysis showed acceptable errors for each of the predicted labels. Synthetic predictions were performed on CPU and took 3.5 min to complete. Additionally, as we had many synthetic events, their predictions were used to demonstrate the reconstruction of subsurface property logs. Figure 7a shows, in magenta, the ground-truth locations associated with synthetics used for the training. In contrast, those associated with pure noise were located at zero depths and zero offsets (in green), as assigned in the training labels. Predicted locations of synthetic and noise patches are shown in blue and green, respectively, and are clearly separated. As a result of including different onset arrival times in the training dataset, the complexity of the neural network model optimization is increased. In turn, this translates into higher variances and biases. This bias is observed as a drift in the predicted depths as offsets increase. The neural network needs to learn from a more diverse dataset to address the bias––in our case, a training dataset that includes more depths. We limited the size of AOI to control the training cost while using limited computing power. In the case of having plenty of predicted events at different depth levels, they can be used to reconstruct subsurface properties, as demonstrated in Figure 7b. Reconstruction of P-wave and S-wave velocities and density at each depth level was conducted by averaging multiple predictions from all offsets. In the case demonstrated in Figure 7, as we only had five depth levels as part of the AOI, each reconstructed property log has five depth values.

### 4.2. Application on CRC-3 Well Field Data

As the synthetics modelling used for the training was based on the vertical geometry CRC-3 well, using a 1D model extended into a 2D model, a more realistic use of the trained model is to detect and predict recorded induced events from the same vertical well. However, this 2D subsurface model still represents the Otway subsurface, which opens the possibility of generalizing the application to other vertical wells in the area.

In this case, the application data was the pre-processed induced seismicity dataset recorded using DAS in the CRC-3 well, composed of 450 patches, most of which were noise patches, and 17 with recorded induced events. As such, reconstruction of subsurface properties was not applicable with a limited number of induced events in the Otway dataset.

**Figure 8:**
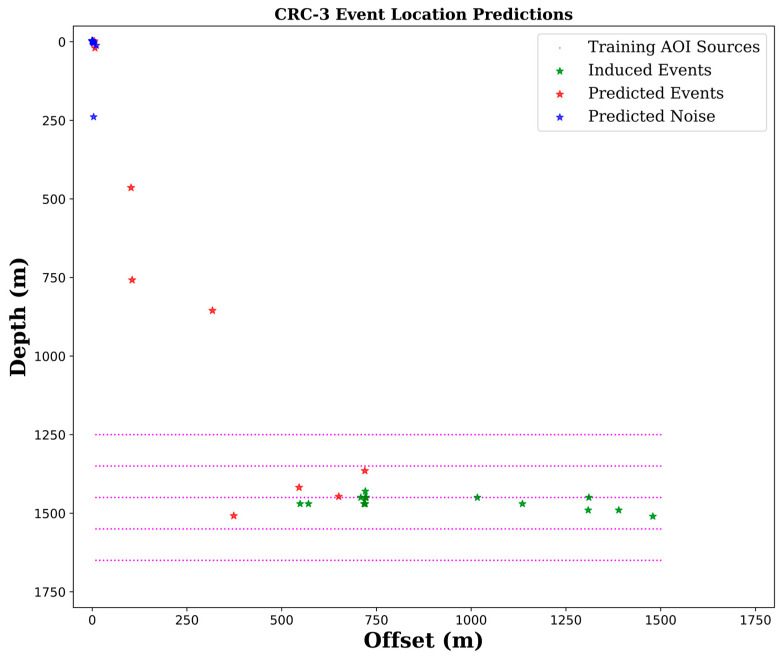
Predictions of event locations for data from vertical CRC-3 well. Green stars represent the actual locations of induced events relative to the well positioned at zero offset. Stars denoting predictions of induced event locations are in red, and predicted noise locations are in blue. The magenta dots show the actual locations of synthetic data used for the training.

Predictions were performed on CPU and took 29.8 s to complete. Figure 8 shows a depth-offset plot of location predictions for data from the CRC-3 well. The green stars represent the actual locations of induced events relative to the vertical well positioned at zero offsets.

Looking at the predicted locations, we observe a clear separation of noise vs. detectable events, marked by blue and red stars, respectively. Most of the predictions of the field data patches containing only noise fell far out of the area of interest at the (x = 0, z = 0) location as expected, with a few exceptions. On the other hand, not all predictions of patches with visually detectible induced events fell within the AOI marked with magenta points. Ten of these events were classified as noise at (x = 0, z = 0) location, and three had a strong bias towards noise. The predictions of four remaining patches fell within the AOI. Unsurprisingly, these four events were strong induced events based on the estimated magnitudes discussed earlier in the data acquisition Section 2.1. Careful examination of these predictions showed reasonable agreement in depth with manual detections marked by green stars.

## 5. Discussion

In this section, we discuss some of the main challenges with our deep learning implementation, how we addressed them, limitations, and potential improvements. First, the problem complexity is discussed, including how different factors add to it by extending the size of the training dataset (e.g., incorporating time shifts). Then, we investigate the effect of different levels of SNR values and acquisition geometry mismatches on the performance of neural networks and possible ways of generalizing their application to other wells.

### 5.1. The Problem Complexity

The complexity of the deep-learning problem stems from the number of variables considered to build an extensive training dataset. Model building starts with variations of 1D models and extends to include different subsurface property parameters and ranges. In the absence of logs or models, multiple model realizations can be generated by randomly perturbing the elastic properties, anisotropy parameters, and the number and thickness of subsurface layers. This, in turn, adds to the problem’s complexity and scale and increases the size of the training data. For simplicity, our implementation is based on the use of 2D models, thus limiting the location predictions to depths and offsets. In reality, in the presence of azimuthal anisotropy, adding an azimuthal term or label would be a possible extension of the implementation to 3D.

Another variable to consider including is different receiver geometries, different well deviations, additional source locations and denser spacing between them. Another factor that could add to the complexity is different source focal mechanisms in synthetic modelling. Typically, in induced seismicity, the source triggering mechanism is not predictable. Recorded induced seismicity events can be of different mechanisms, which can add valuable information on how they were triggered, but do not contribute to detecting them. Different source mechanisms can be simulated for each source location, yielding similar wave propagation kinematics but different radiation patterns. We use a vertical point source to limit the modelling perturbations and correct radiation patterns of the prediction data in the preparation workflow. Other factors related to the triggering time and the strength of the source are uncontrollable for passive measurements. The strength of the source is directly related to the signal-to-noise ratio of recorded data. Considering all these variables can inflate the training dataset’s size and increase the number of scenarios a model needs to learn, thus increasing the training time and the required computing resources.

The size and, hence, sufficiency of the training dataset is an often-overlooked issue. Generally, a practical way of understanding data sufficiency and selection of specific deep-learning models is by looking at their performance and learning curves [27,28]. In other words, it is a balance between the size of the training dataset and the model’s performance. The number of variables a deep-learning model needs to learn during training contributes directly to the complexity of the model. Furthermore, the more complex the model is, the harder it is to optimize through training. Using a small training dataset yields low training errors but high validation errors, indicating overfitting. In other words, the model learns the small dataset very well but fails to predict new data. On the other hand, as we increase the training data size, the validation errors decrease as the model tends to generalize better from a higher amount of data. However, there will be a point where increasing the size of the training dataset will only increase the training time and not contribute to better performance. In our case, we include some of these variables, such as time shifts and variable SNR values, and investigated their effects while neutralizing the effects of other variables within the data preparation workflows.

### 5.2. Effects of Time-Shifts

**Figure 9:**
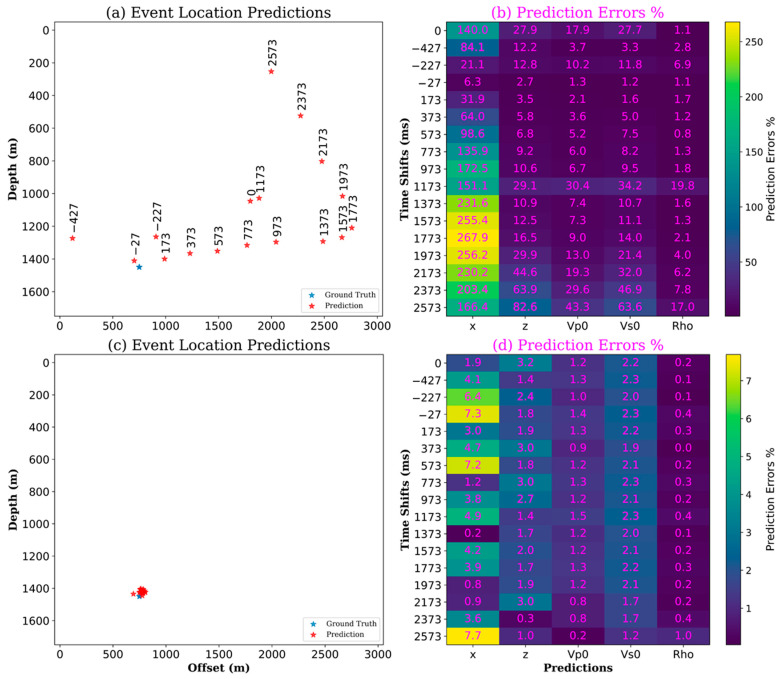
The effect of not including time shifts in the training data on (**a**) event location predictions with time-shift annotations, and (**b**) the resulting large prediction errors, as compared to the effect of including time shifts in the training data on (**c**) event location predictions, and (**d**) the resulting reasonably low prediction errors.

One of the challenges of using convolutional neural networks trained on synthetic data on passive data is the onset time mismatch. This mismatch is represented in the unknown onset time of the passive data. To demonstrate the effect of this mismatch on neural network inferences, a single synthetic event was selected, initially shifted by multiples of 200 milliseconds, resulting in six patches of the same event with six different time shifts. This small dataset was then used for event location prediction using a trained neural network model that did not include time shifts in the training data, as shown in Figure 9a. Subsequently, prediction errors were calculated and are shown in Figure 9b.

The same test was repeated with a second event shifted by multiples of 200 milliseconds. This dataset was then used for event location prediction using a second trained model that included time shifts in the training data, as shown in Figure 9c. Subsequently, prediction errors were calculated and shown in Figure 9d. The first model shows that the neural network is sensitive to time shifts in the input images, resulting in larger prediction errors as time shifts increase.

Another observation is that predictions of offsets have larger errors than the rest of the predicted properties, probably attributed to offsets being less correlated to depth and other predictions. On the other hand, introducing time shifts in training neural network models generates much lower errors. This observation prompted us to consider extending the training dataset to include time shifts.

### 5.3. Effects of Signal-to-Noise Ratio

**Figure 10:**
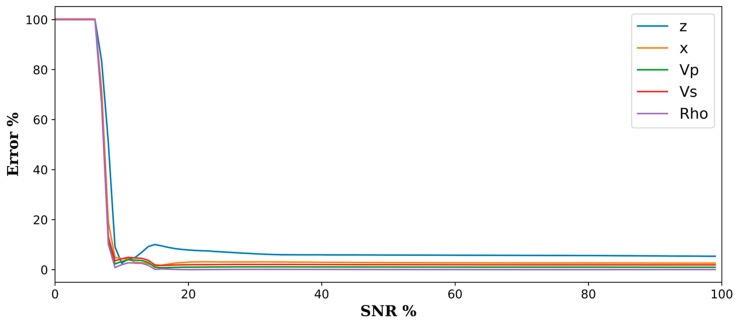
The effect of varying synthetics signal-to-noise ratios on detection.

An inherent limitation of convolutional neural networks is their lower performance at low SNR values [29]. We tested this for the ResNet50 architecture by generating 100 patches combining a single synthetic event with the same noise record. A scalar multiplier varied the signal level between 0 and 1. These patches were then fed into the trained neural network for predictions. As known labels were also generated for this small dataset, errors were calculated for each of the predictions, as shown in Figure 10. For SNR values less than nine percent, predictions have huge errors and stabilize above that. The ResNet50 architecture performance was the worst compared to other network architectures, which led to the consideration of other architectures, such as AlexNet, to improve the detectability of induced events of low SNR values. This led us to another consideration of including denoising as part of our implementation, particularly in the data preparation workflow [30,31].

### 5.4. Application to Other Wells Field Data

**Figure 11:**
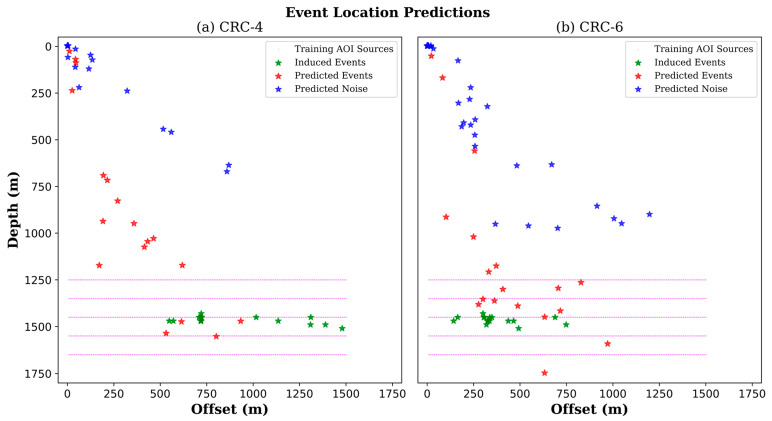
Predictions of event and noise locations for data from slightly deviated (**a**) CRC-4 and (**b**) CRC-6 wells. Green stars represent the actual locations of induced events relative to the well positioned at zero offset.

In this subsection, we explore the generalization of the application to other nearby wells with slightly deviated geometries, namely CRC-4 and CRC-6, located 15 and 1015 m away from the CRC-3 well, respectively. However, the application using data from deviated wells was not ideal, as their geometries were not represented in the dataset used for training. Recorded data from CRC-4 and CRC-6 wells were prepared with the same workflows, which resulted in 450 patches for each well. Then, they were fed into the neural network for predictions. Location predictions associated with event patches for both CRC-4 and CRC-6 wells have relatively reasonable separation from those related to noise, as shown in Figure 11a,b. However, patches associated with noise were less focused on the zero-location compared to CRC-3 predictions.

For CRC-4 event location predictions, the same four strong events were detected or classified within the expected AOI. However, fewer patches, including events, were classified as pure noise and fell further away from the (x = 0, z = 0) location. Ten of these patches were closer to the AOI. It is worth mentioning that CRC-4 data had better SNR than CRC-3 data, which may explain why more patches fell closer to the AOI. For CRC-6 event location predictions, more event patches fell within the AOI, and most of the rest were further away from the noise (x = 0, z = 0) location. This is attributed to the higher SNR of the CRC-6 data than the CRC-3 and CRC-4 data, mainly as most induced events occur closer to the CRC-6 well.

## 6. Conclusions

In this article, we explored and evaluated a supervised deep-learning approach for detecting and localizing passive seismic sources and demonstrated using them to reconstruct subsurface properties. We also identified the challenges of using supervised deep-learning neural networks, particularly the reliance on synthetic data for training. To address these challenges, we developed and implemented data preparation workflows. The methodology was validated using synthetic datasets, which indicated significant potential for application to passive seismic data. In scenarios with a large number of passive events, the approach can be used to reconstruct subsurface property logs.

Further testing with synthetic data examined the neural network’s sensitivity factors, such as time shifts, varying signal-to-noise ratio (SNR) values, and geometry mismatches. Accounting for these factors improved the network’s performance, yielding lower error rates. We then applied the methodology to the induced seismicity dataset from the Otway CO_2_ injection site. The results on the CRC-3 well data demonstrated excellent noise versus signal classifications, benefiting from training on data modelled with CRC-3 geometry. However, the effects of suboptimal distributed acoustic sensing (DAS) data quality in the CRC-3 well highlighted the inherent limitations of convolutional neural networks at low SNR values.

Application on other wells underscored the importance of matching acquisition geometries when modelling training data. These findings provide valuable insights, paving the way for more comprehensive studies incorporating additional variables to produce more representative training data, thus further bridging the gap between synthetic and field passive seismic data. Ultimately, this approach enhances the detection of induced seismic events across multiple wells and opens up possibilities for broader applications, such as seismic while drilling (SWD).

In the end, we discussed the main enablers and challenges of the approach and how each contributed to the discussed overall problem complexity. These discussions also explained potential future expansions of the method, considering more variables in the synthetic modelling and multi-stage prediction approaches.

## Data Availability

Restrictions apply to the availability of these data. Data were obtained from the CO2CRC project and are available to its members. The membership can be requested at info@co2crc.com.au.
